# Identification of research gaps to improve care for healthy ageing: a scoping review

**DOI:** 10.1136/fmch-2024-003116

**Published:** 2024-10-23

**Authors:** Matteo Cesari, Marco Canevelli, Jotheeswaran Amuthavalli Thiyagarajan, Soung-Eun Choi, Polina Grushevska, Saloni Kumar, Muyan Chen, Hyobum Jang, Yuka Sumi, Anshu Banerjee

**Affiliations:** 1 Ageing and Health Unit, Department of Maternal, Newborn, Child and Adolescent Health and Ageing, World Health Organization, Geneva, Switzerland; 2 Department of Neurobiology, Care Sciences and Society, Stockholm University, Stockholm, Sweden; 3 National Center for Disease Prevention and Health Promotion, Italian National Institute of Health, Rome, Italy; 4 Department of Human Neuroscience, Sapienza University, Rome, Italy; 5 Master in Health and International Development, London School of Economics, London, UK; 6 Department of Maternal, Newborn, Child and Adolescent Health and Ageing, World Health Organization, Geneva, Switzerland

**Keywords:** Aging, Community Medicine, Environment and Public Health, Quality of Health Care, Global Health

## Abstract

**Objective:**

Several research gaps affect the improvement of care for healthy ageing. Their identification is crucial to developing a specific research prioritisation agenda supporting progress at the micro (clinical), meso (service delivery) and macro (system) levels. To achieve this, a scoping review was carried out to describe the most significant gaps impeding the improvement of care for healthy ageing.

**Design:**

A scoping review of the literature was conducted according to the Joanna Briggs Institute methodology. The selected articles were analysed to identify topics or areas essential for improving care for healthy ageing but requiring further support from research.

**Eligibility criteria:**

Every type of scientific article, except for randomised controlled trials, was considered of potential interest without restrictions on publication date, type of publication and methodology.

**Information sources:**

A systematic search (last search: 6 December 2023) was conducted using PubMed, MEDLINE and Scopus.

**Results:**

Overall, 1558 articles were retrieved from the literature. Of these, 310 were finally retained for this work. A total of 1195 research gaps were identified (average: 3.85 per article) and clustered into the 13 primary areas: ageing, care approach, caregivers, health economics, health, interventions, policies, research, settings, training, technology, specific populations and understanding the older person. In particular, research for improving the person-centred approach (n=38), better considering cultural diversities (n=27), implementing integrated care (n=25) and ensuring access to care (n=25) were the most prevalent priorities reported in the literature.

**Conclusions:**

A wide range of factors spanning multiple disciplines, from clinical to policy levels, require special consideration, exploration and resolution. The findings of this scoping review represent an essential step in identifying gaps for developing a research prioritisation agenda to improve care for healthy ageing.

**WHAT IS ALREADY KNOWN ON THIS TOPIC**

- Several research gaps affect the improvement of care for healthy ageing. Their identification will allow to develop a consistent research prioritisation agenda.

**WHAT THIS STUDY ADDS**

- The analysis of the available literature identified multiple research areas requiring special consideration, from the mechanisms of ageing to health economics, from the need for a better understanding of the older person to reorientating care towards novel standards.

**HOW THIS STUDY MIGHT AFFECT RESEARCH, PRACTICE OR POLICY**

- The findings of this scoping review represent an essential step in developing a research prioritisation agenda aimed at improving care for healthy ageing. A global, multistakeholder collaboration is required to promote high-quality care for older persons.

## Introduction

 Globally, every country is experiencing important demographic and epidemiological changes. As people age, their likelihood of developing chronic conditions increases.^1 2^ Nonetheless, the challenges faced by older individuals with chronic conditions cannot be solely attributed to the conditions themselves.^3^ To comprehensively address the health and care needs of individuals, a shift is needed from a disease-centred approach to a holistic perspective that considers the person’s capacities, abilities, values and priorities throughout the life course.^3^ This shift necessitates personalising care and restructuring health and social care systems to better allocate resources and interventions for the ageing population.^4^

In this context, it is noteworthy that, in 2020, the United Nations (UN) declared the Decade of Healthy Ageing (2021–2030) with support from the WHO.^5^ Various stakeholders are encouraged to act towards the improvement of the lives of older people, their families and the communities.^6 7^ Action areas include delivering person-centred, integrated care and providing access to long-term care for older people who need it. This integration and personalised interventions aim to make our systems better responsive to the needs of the older population.^8^

Several research issues are currently affecting the improvement of care for healthy ageing. These include the lack of age-disaggregated data,^9 10^ limited evidence from longitudinal studies,^7^ a monodimensional approach to ageing^11^ and age-related conditions,^12^ and the frequent exclusion of older persons from clinical trials due to too stringent eligibility criteria.^13^,^15^ To facilitate progress at the micro (clinical), meso (service delivery) and macro (system) levels, it is crucial to develop a specific agenda that identifies and organises current knowledge gaps for prioritising research needs. Indeed, this review is crucial to a larger, long-term project to set research priorities. Identifying these priorities is important for two main reasons: first, to improve service delivery for enhancing individuals’ intrinsic capacity and functional ability, and second, to timely and efficiently address the unmet needs of older people, especially those facing complex clinical, sociocultural and environmental challenges.

As mentioned, this work presents the results of a scoping review designed to identify the most significant and urgent research gaps in the scientific literature that hinder the advancement of care for healthy ageing. The present review will be used as essential background material to initiate and inform a project, coordinated by the WHO and expected to be completed in 2025, to develop a research prioritisation agenda. In particular, based on an adapted Delphi methodology, a multistep expert consensus process will lead to identifying and agreeing with research priorities to improve care for healthy ageing, considering public health benefits, feasibility and costs.

## Methods

### Study design

The scoping review method was chosen given the novelty and vastity of the topic (ie, research gaps to improve care for healthy ageing). The purpose was to understand the current state of research in the field and identify the research gaps in the most comprehensive and exhaustive way. In the present work, a research gap was defined as ‘a topic or area for which missing or inadequate information limits the ability […] to reach a conclusion for a given research question’.^16^ The scoping review was conducted according to the Joanna Briggs Institute methodology for scoping reviews^17^ and reported following the Preferred Reporting Items for Systematic Reviews and Meta-Analyses extension for scoping reviews checklist.^18^

### Patient and public involvement

Our work, based on exploring available literature, aligns with the Plan of Action for the UN Decade of Healthy Ageing (2021–2030). It represents an essential preliminary step of a Delphi survey coordinated by the WHO to develop a research prioritisation agenda for improving care for healthy ageing through the involvement of a broad panel of stakeholders, including older persons and their families.

### Search strategy

A systematic search of the peer-reviewed literature published from inception to 6 December 2023 was conducted using PubMed, MEDLINE and Scopus. These databases were selected to source multidisciplinary literature on the social and medical aspects of care for older persons. The search strategies were formulated with the assistance of a skilled librarian. The final search results were extracted and imported into Covidence, a specialised software for literature review management. Duplicates were removed.

Search terms were negotiated and approved collaboratively by researchers and referred to the following three areas of interest: (1) healthy ageing, (2) care and 3) gaps/priorities. The Boolean operator “OR” was used to combine search terms within each interest area, whereas “AND” was used to combine the three interest areas (online supplemental file 1).

### Selection of articles

An article was considered of interest for defining research gaps to improve care for healthy ageing when it explicitly reported them in the form of sentences or paragraphs. These could relate to every aspect of care, from design to its organisation, from service delivery to specific conditions.

Every type of scientific article published in English was considered of potential interest without restrictions on publication date, type of publication (eg, original article, reviews and consensus articles) and methodology (eg, qualitative studies and quantitative studies), except randomised controlled trials. This choice was motivated by the fact that participants in randomised controlled trials are not usually representative of the general older population. At the same time, the rigid objectives of trials could implicitly determine a partial and biased vision in defining research gaps and priorities.

Six reviewers screened titles and abstracts of all the articles retrieved through the literature search to filter potentially relevant studies. An agreed conservative approach was used at this preliminary step. Accordingly, articles were kept for more in-depth consideration of the full text if they had the potential to contain relevant sentence(s) on the topic of interest.

In a subsequent step, pairs of reviewers independently screened the full texts of all potentially eligible studies. Any discrepancy was discussed and resolved by consensus; if this was not possible, a third reviewer was involved in deciding whether to include the article in the final set of publications to analyse.

### Data charting process

Data from the retained studies were extracted using Covidence and exported to an ad hoc generated and shared Microsoft Excel file. Six reviewers charted the data and collaboratively populated the data-charting form. Every extracted article was distinguished by a unique identifier.

The following information was extrapolated from the retrieved studies: (1) journal name, (2) year of publication and (3) every sentence in the article identifying a research gap or priority to improve care for healthy ageing.

### Synthesis of results

For each article, two researchers extracted the most meaningful and representative keywords or long-tail keywords for every sentence that referred to a possible research gap or priority. These keywords were then listed horizontally in the data-charting form. It is important to note that a single sentence could generate multiple keywords. For instance, a sentence like ‘more research is needed to understand the needs of older persons with HIV, especially in low-resource settings’ could generate keywords such as “HIV”, “low-resource setting” and “understanding of needs of older persons”. After conducting a vertical analysis of the keywords, they were discussed and made more standardised and consistent (eg, by removing synonyms).

The resulting homogenised keywords were then added to a mind map created using a specific online tool (ie, Wisemapping; https://www.wisemapping.com/). The keywords were then tentatively organised into clusters in the mind map. During the clustering process, the researchers engaged in discussions that were aimed at coherently representing the identified gaps in the mind map as meant in the original source. This was done by also going back to the specific article to better interpret the meaning of the keywords and the context from where they were extracted. At the same time, researchers also tried to ensure the most comprehensive representation of the evidence while synthesising and incorporating the topics to avoid the dispersion brought by marginal or isolated themes.

## Results

**Figure 1:**
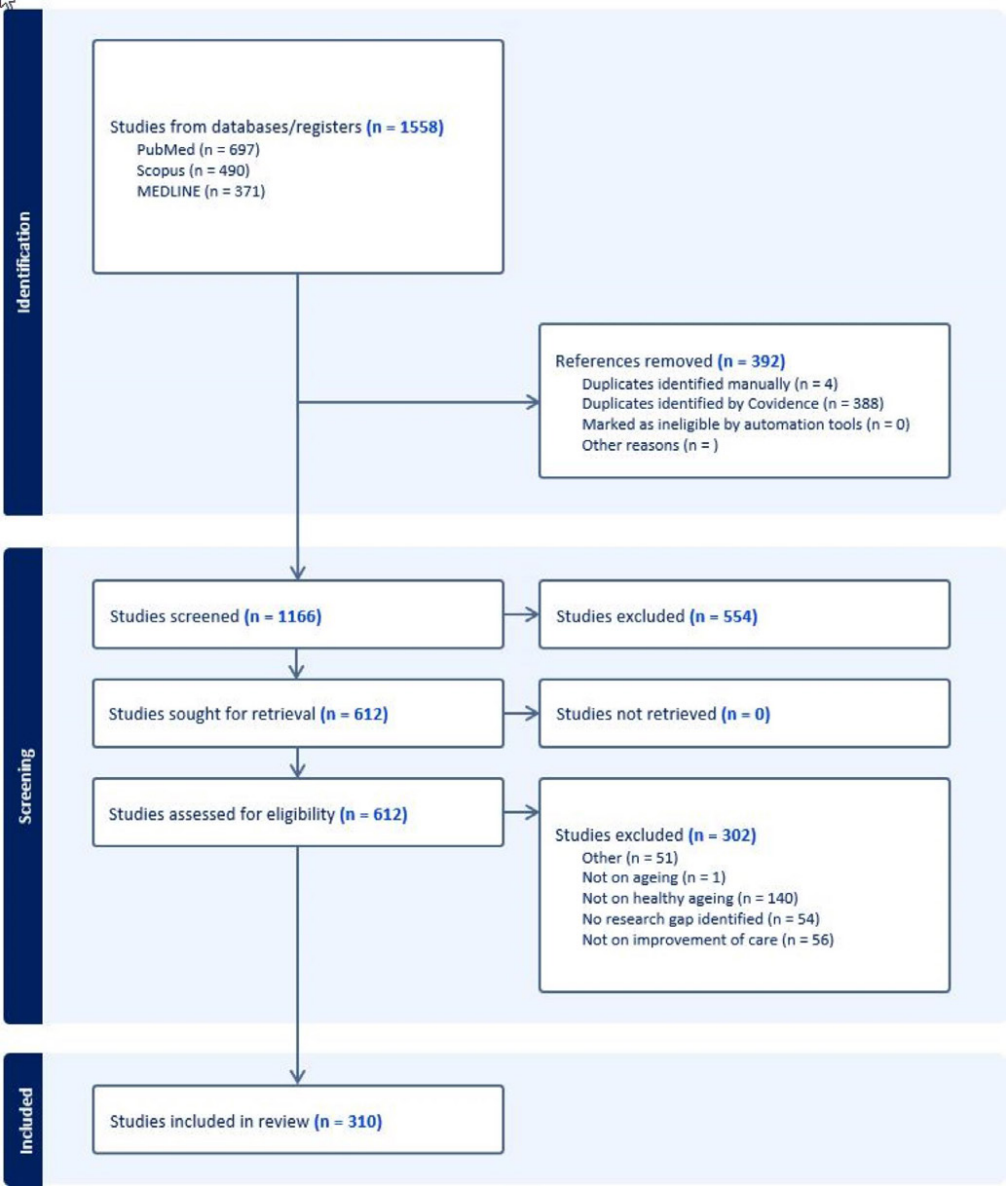
Flow chart describing the selection process of articles of interest for the scoping review.

Figure 1 presents the results of the search strategy adopted in this scoping review. Overall, 1558 articles were retrieved from PubMed (n=697), Scopus (n=490) and MEDLINE (n=371). Of these, 1166 underwent a first screening, after 392 were removed as duplicates. Further 554 articles were additionally excluded from the in-depth analysis as their titles and/or abstracts were clearly not of interest to the purpose of the scoping review. Among the residual 612 articles assessed for eligibility through full-text evaluation, 310 were finally retained for the present work. The list of the articles finally retained for the identification of research gaps is available in online supplemental file 2. The research gaps identified in each article are provided.

**Figure 2:**
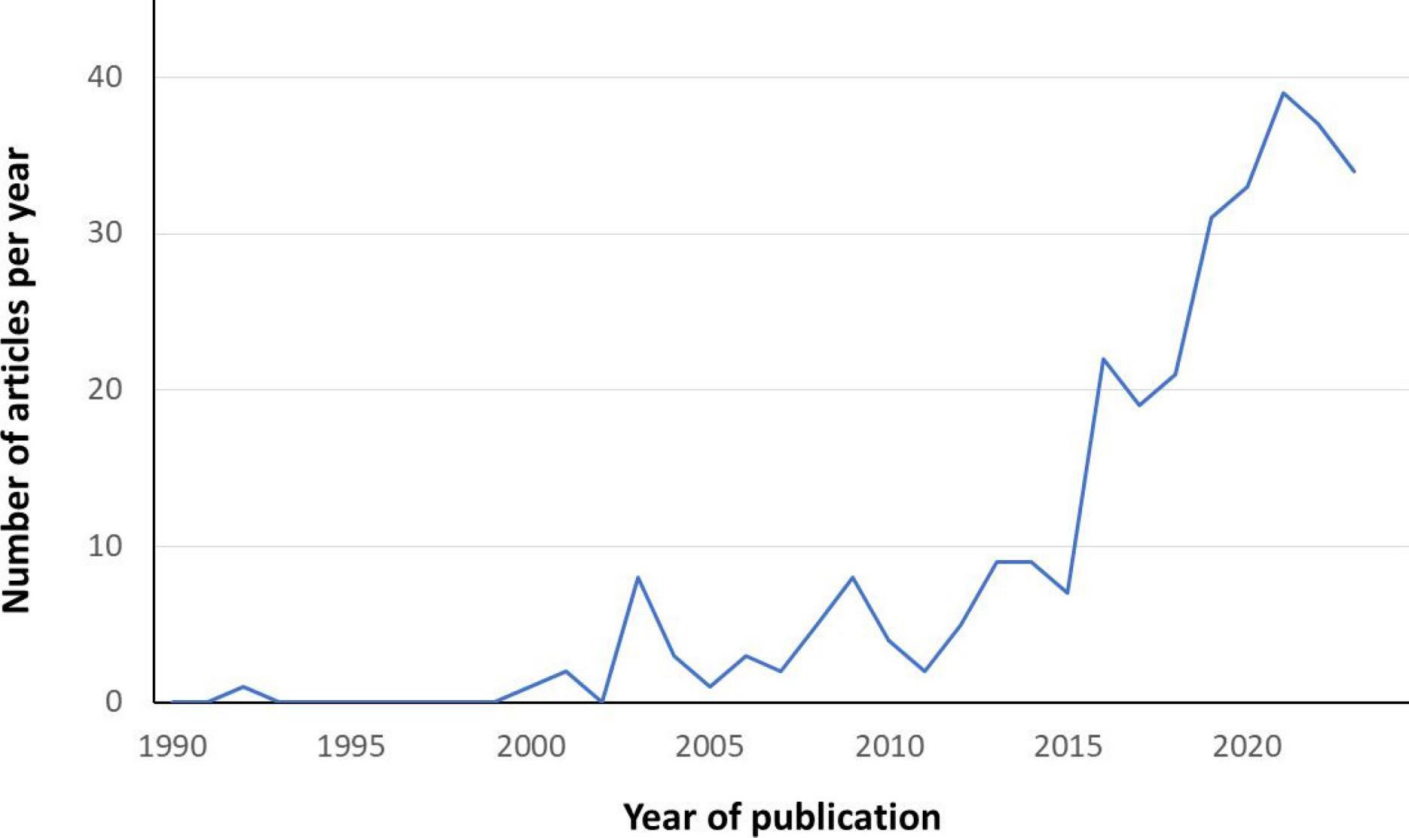
Number of articles finally retained for the scoping review per year of publication.

**Figure 3:**
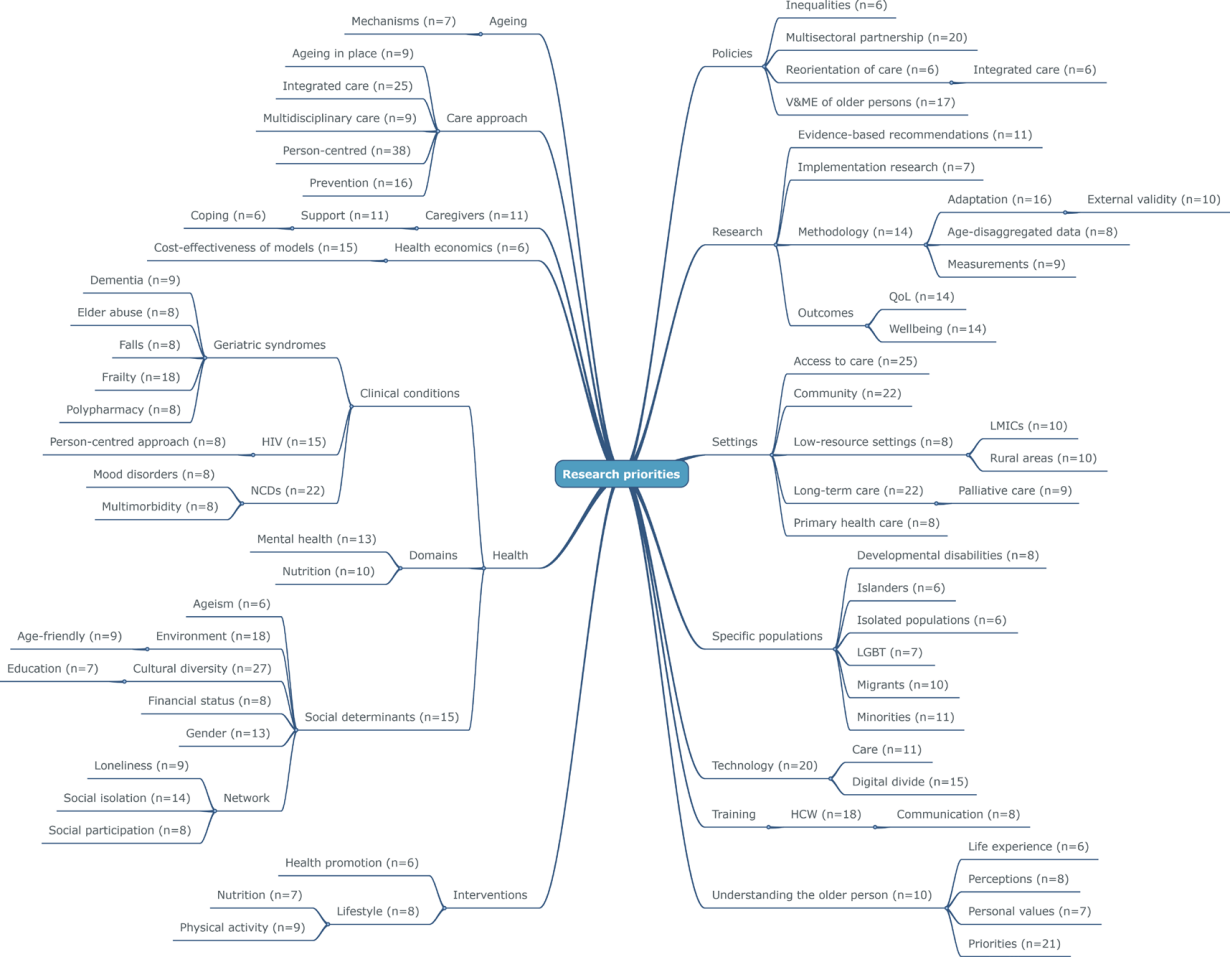
Simplified mind map of cluster and subclusters of research gaps as identified by the scoping review. Only the most prevalent research gaps (reported more than five times) are represented. The number of entries for each research gap is indicated in parentheses. HCW, healthcare worker; LGBT, lesbian, gay, bisexual, transgender; LMICs, low-income and middle-income countries; NCDs, non-communicable diseases; QoL, quality of life; V&ME, voice and meaningful engagement.

Overall, the number of publications in the field has increased exponentially over the past 10 years (figure 2). By reviewing the full texts of the 310 articles of interest, 1195 research gaps were identified (an average of 3.85 per article). Figure 3 presents the final, simplified output of the present scoping review and visualises the organisation of the most prevalent research gaps into thirteen clusters (ie, ageing, care approach, caregivers, health economics, health, interventions, policies; research, settings, training, technology, specific populations and understanding the older person) and the most relevant subclusters. A detailed presentation of the results is available in online supplemental file 3.

### Ageing

This first cluster included articles referring to the need for more research on the various mechanisms of ageing (n=7) to better understand the pathophysiological changes underlying age-related conditions. The cluster also included articles soliciting work to improve/disseminate the narrative related to ageing.

### Care approach

This cluster of research gaps encompasses various methodologies and dynamics characterising a care approach that promotes healthy ageing and/or is responsive to the needs of older persons. For instance, many entries referred to the importance of adopting a person-centred approach (n=38), integrating care (n=25), having a preventive approach in the design, organisation or delivery of care (n=16), fostering multidisciplinary collaborations (n=9) and enabling ‘ageing in place’ (n=9).

### Caregivers

This set of research gaps underscores the need for additional research on the roles, priorities and activities of caregivers. The findings indicate that greater effort is needed on how to support caregivers (n=11). Moreover, further investigation is warranted to understand the coping dynamics and strategies employed by caregivers in their specific and unique situations of care burden (n=6).

### Health economics

The cluster on ‘health economics’ primarily focuses on conducting cost-effective analyses to adequately support interventions and strategies for promoting healthy ageing (n=15). It emphasises the importance of verifying the cost-effectiveness of actions and interventions to better allocate care resources.

### Health

This cluster includes all the entries concerning a wide range of research gaps referring to older persons’ physical, mental and social conditions. The first subcluster covers specific clinical conditions such as geriatric syndromes (ie, frailty (n=18), dementia (n=9), elder abuse (n=8), falls (n=8), and polypharmacy (n=8)), non-communicable diseases (NCDs; n=22), and HIV (n=15). In particular, it highlights the need for more research on how to personalise care for persons living with HIV (n=8) and, among NCDs, mood disorders (n=8) and multimorbidity (n=8).

A second subcluster grouped all the health domains indicated as critical for healthy ageing. Nutrition (n=13) and mental health (n=10) emerged more consistently.

Finally, a third subcluster was composed of research gaps referring to social determinants of health, including cultural diversity (n=27), environment (n=18), gender (n=13), financial status (n=8), education (n=7) and ageism (n=6). In this context, numerous entries pointed to the need for better work on the social network of older persons, especially on the conditions of social isolation (n=14) and loneliness (n=9).

### Interventions

Gaps and priorities that require more research on specific interventions, such as lifestyle interventions (n=8) and health promotion (n=6), are included in this cluster. Regarding lifestyle modifications, special attention was given to physical activity (n=9) and nutrition (n=7).

### Policies

Several articles indicated the need for more research activities for the design, development and adoption of policies promoting better care for older persons. In particular, it mentioned the need for activities aimed at promoting multisectoral partnership (n=20) and the meaningful engagement of older persons (n=17). Policies were also indicated as critical to address inequalities (n=6) and facilitate the reorientation of care towards more integrated models (n=6).

### Research

This cluster includes entries that outline specific activities for conducting research to improve care for older persons. Many of them emphasise the need for more evidence-based recommendations (n=11) and call for more implementation research (n=7). Other entries focus on specific methodological aspects and shortcomings (n=14), such as the need to adapt methodologies (n=16), enhance the external validity and generalisability of the findings (n=10), improve the currently poor sensitivity of measures (n=9) and increase the visibility of older persons in research (ie, age disaggregation, n=8). Quality of life (n=14) and well-being (n=14) were frequently highlighted as specific outcomes to prioritise in research activities for older persons.

### Settings

The research gaps included in this category focus on the need to find solutions to improve access to care for older persons (n=25). Entries also indicated the importance of conducting more research in specific contexts where older persons may seek care, including the community (n=22) and low-resource settings (n=8), in particular low-income and middle-income countries (n=10) and rural areas (n=10).

### Training

This cluster groups entries indicating the necessity to improve the training of health and care workers to better cater for the specific needs and priorities of older persons (n=18). The cluster includes eight entries emphasising the significance of improving communication strategies used by health and social care workers when interacting and conversing with older persons (n=8).

### Technology

A significant proportion of the content grouped in this cluster focuses on the need for further research on technologies for promoting healthy ageing. Specific areas of work should be on the use of technologies in healthcare delivery (n=11) and addressing the digital divide often experienced by older persons (n=15).

### Specific populations

Several entries highlighted the importance of addressing subgroups of older persons who are considered more vulnerable and/or have not been adequately considered in existing research. In particular, more research was requested to comprehend the unique healthcare needs and challenges faced by minorities (n=11), migrants (n=10), individuals with developmental disabilities (n=8), the lesbian, gay, bisexual, transgender (LGBT) community (n=7), people living on islands (n=6) and isolated groups (n=6).

### Understanding the older person

This cluster encompasses several entries related to the challenges of understanding the older person (n=10). It was repeatedly reported the importance of more research on how to capture and consider the priorities (n=21), perceptions (n=8), personal values (n=7) and life experiences (n=6) that the older person may have and that are crucial in providing meaningful care.

## Discussion

This scoping review aims to identify and categorise the research gaps impeding the improvement of care for healthy ageing, as highlighted in the scientific literature. Our findings indicate a range of factors spanning across multiple disciplines, from clinical to policy levels, which require further consideration, exploration and resolution. This underscores the necessity for collaborative and multisectoral actions, as outlined in the declaration^5^ and plan of action^19^ of the UN Decade of Healthy Ageing (2021–2030).

Establishing research priorities can illuminate areas where more evidence is urgently needed to make informed decisions and improve practices. Developing a research prioritisation agenda can foster collaborations and create opportunities for investment and growth. Furthermore, by identifying research gaps, the agenda can prioritise actions to efficiently achieve broader goals.

The agenda will have to take into account the following factors:

1. The heterogeneous development stage and organisation of the health and care systems globally.
2. The involvement of a wide range of stakeholders.
3. The complexity of the needs, priorities and preferences of the older population.
4. The different contexts, determinants and values across countries and societies that impact the dynamics of health and social care systems.

However, developing a research prioritisation agenda requires some background material to initiate discussions among multiple stakeholders, especially in a vast and complex field such as care for older persons. In this context, the present scoping review serves as a preliminary step to describe the existing research gaps. This work complements and is an extension to a report we recently published on the same topic,^20^ which describes the results of a survey conducted with a panel of international experts. These two articles will be used as the necessary background material to initiate an adapted Delphi survey with wide stakeholder involvement, including older people and family members,^7^ towards a comprehensive discussion and prioritisation of research gaps.

It is noteworthy to observe disparities between the insights provided by experts and the findings uncovered in the present scoping review. The review covers a wider spectrum of research gaps and clusters, likely due to the more significant number of inputs considered. At the same time, the survey results may have been influenced and biased by participants’ familiarity with the WHO framework of healthy ageing, whereas the scoping review implicitly provides a more comprehensive view. In both documents, the need for more research on the personalisation of care is well evident. The importance of promoting a person-centred approach to older persons is clearly visible in the scoping review, being the most prevalent entry reported in the scientific literature. This need is also reflected in the cluster, which is common in both works, pointing to the importance of better understanding the priorities of older persons. The previous report indicated the need for more research in low-resource settings, which is confirmed and extended by the scoping review. In fact, the present results suggest the opportunity to specifically promote research in low-income and middle-income countries as well as in rural areas. Additionally, the scoping review highlights the importance of studying subgroups of older people that are often under-represented in scientific literature, such as migrants, minorities and isolated populations.

Our findings also emphasise the need to reconsider the methodology of research conducted on older persons. It is suggested that research goals, design and measurements should be adjusted to produce recommendations that can be translated into a sustainable and acceptable practice. In this context, the experts involved in the previous survey recommended giving more weight to qualitative and pragmatic studies. The scoping review also underlines the necessity for research that is closer to implementation and the use of outcomes that are more meaningful for the individual (eg, quality of life and well-being).

Likely due to its more comprehensive approach, the scoping review reported a wider range of clinical conditions that should be studied in older persons, not limited to NCDs and geriatric syndromes. Our findings show that care for persons living with HIV, a condition that has been associated with accentuated and accelerated ageing,^21^ needs improvement. In particular, the entries highlighted the importance of expanding the focus of care beyond just the early initiation of the treatment and the reduction of the viral load to more responsively address the diverse needs of the person. Many studies included in the final analyses of the scoping review emphasised the need for more research on the social determinants of health, especially cultural diversity, social networks and gender.

Finally, it is noteworthy that most articles included in our analysis were published over the past 10 years despite our comprehensive and inclusive approach to the literature. This was primarily explained by the exponentially increasing but relatively recent interest of the scientific community in healthy ageing.

The present study has some limitations that are worth mentioning. Despite our efforts to comprehensively map research gaps in the scientific literature, some might have been inadequately represented or missed. For example, we limited our search strategy to the main databases of scientific literature, excluding the analysis of the so-called ‘grey literature’. This strategy was motivated to ensure that the research gaps we identified were from reliable and recognised sources of evidence, reducing the risk of including non-scientific or inadequately proven entries in our analyses. At the same time, our approach may have underestimated research gaps from settings, disciplines and stakeholders that are not adequately represented in the scientific literature. We also acknowledge the possibility of errors during the process of standardisation and clustering of research gaps. To mitigate the risk, we took several precautions, such as having multiple independent reviewers analyse the sources, engaging in iterative discussions to reach consensus and re-evaluating the original text in case of uncertainties. The high number of entries we analysed should have further contributed to substantially minimising this issue. A further limitation can be identified in our decision not to consider research gaps proposed in articles reporting the results of randomised controlled trials. Although a randomised controlled trial is essential for generating evidence, its role in defining research gaps may be arguable. In fact, randomised controlled trials are designed to answer a research question monodimensionally. The adopted methodology (including the formulation of the research question) may impact the subsequent results, potentially influencing the conclusions. Pragmatic trials might be an exception, but their use, as suggested by some of our findings, is still very limited.^22^22

In conclusion, the findings of this scoping review are an essential step in identifying gaps and priorities for developing a research agenda to improve care for healthy ageing. Multiple diverse research gaps currently hinder the ability to provide high-quality care to older persons, implicitly calling for urgent multisectoral and collaborative actions. In this context, the development of a research prioritisation agenda with the involvement of a broad spectrum of global stakeholders is critical to promote healthy ageing.

## supplementary material

10.1136/fmch-2024-003116 online supplemental file 1

10.1136/fmch-2024-003116 online supplemental file 2

10.1136/fmch-2024-003116 online supplemental file 3

## Data Availability

All data relevant to the study are included in the article or uploaded as supplementary information.
