# Supplementary material for: Identification of research gaps to improve care for healthy ageing: a scoping review
Source: Fam Med Community Health. 2024 Oct 23;12(4):e003116. doi: 10.1136/fmch-2024-003116 (PMC11499781; doi:10.1136/fmch-2024-003116)
Supplement: online supplemental file 1 [file fmch-12-4-s001.pdf]

Annex 1. Keywords used to search the literature of interest.

OR	Healthy aging	Care	Agenda
	Healthy ageing	Improve care	Research gap
	Successful aging	Improve of care	Research priorit*
	Successful ageing		Gap*
	Active aging		Priorit*
	Active ageing		Barrier*
	Aging well		
	Ageing well		
	AND		
