# Supplementary material for: Identification of research gaps to improve care for healthy ageing: a scoping review
Source: Fam Med Community Health. 2024 Oct 23;12(4):e003116. doi: 10.1136/fmch-2024-003116 (PMC11499781; doi:10.1136/fmch-2024-003116)
Supplement: online supplemental file 3 [file fmch-12-4-s003.pdf]

Annex 2. Cluster and subclusters of research gaps as identified by the scoping review. The number of entries for each research gap is reported in parentheses.

1. Ageing
    1.1. Framework (n=2)
        1.1.1. Benefits (n=2)
        1.1.2. Definition (n=5)
        1.1.3. Life-course (n=5)
        1.1.4. Resilience (n=3)
    1.2. Mechanisms (n=7)
        1.2.1. Biology (n=5)
        1.2.2. Trajectories (n=5)
2. Care approach
    2.1. Ageing in place (n=9)
    2.2. Decision-making process (n=3)
    2.3. Integrated care (n=25)
        2.3.1. Care pathways (n=4)
    2.4. Life-course (n=5)
    2.5. Multidisciplinary care (n=9)
        2.5.1. Nursing (n=3)
    2.6. Person-centred (n=38)
    2.7. Prevention (n=16)

2.7.1. Self-care (n=3)

2.8. Traditional medicine (n=5)

3. Caregivers (n=11)

3.1. Informal (n=3)

3.2. Support (n=11)

3.2.1. Coping (n=6)

3.3. Wellbeing (n=3)

3.3.1. Care recipients (n=3)

4. Health economics (n=6)

4.1. Cost-effectiveness of models (n=15)

5. Health

5.1. Clinical conditions

5.1.1. Geriatric syndromes (n=3)

5.1.1.1. Dementia (n=9)

5.1.1.2. Disability (n=4)

5.1.1.2.1. Prevention (n=4)

5.1.1.3. Elder abuse (n=8)

5.1.1.4. Falls (n=8)

5.1.1.5. Frailty (n=18)

5.1.1.6. Pain (n=4)

5.1.1.7. Polypharmacy (n=8)

5.1.1.7.1. Prescribing (n=3)

- 5.1.2. Infectious diseases (n=2)
  - 5.1.2.1. HIV (n=15)
    - 5.1.2.1.1. Ageing (n=3)
      - 5.1.2.1.1.1. Clinical complexity (n=5)
        - 5.1.2.1.1.1.1. Mental health (n=3)
      - 5.1.2.1.1.2. Integrated care (n=4)
      - 5.1.2.1.1.3. Low-resource settings (n=2)
      - 5.1.2.1.1.4. Person-centred approach (n=8)
      - 5.1.2.1.1.5. Stigma (n=3)
- 5.1.3. NCDs (n=22)
  - 5.1.3.1. Cancer (n=3)
  - 5.1.3.2. Mood disorders (n=8)
  - 5.1.3.3. Multimorbidity (n=8)
  - 5.1.3.4. Musculoskeletal disorders (n=4)

5.2. Domains

- 5.2.1. Mental health (n=13)
  - 5.2.1.1. Psychological (n=5)
  - 5.2.1.2. Sleep (n=2)
- 5.2.2. Nutrition (n=10)
- 5.2.3. Oral health (n=5)
- 5.2.4. Physical
  - 5.2.4.1. Balance (n=3)

5.2.4.2. Functional status (n=5)

5.2.4.3. Mobility (n=2)

5.2.5. Sensory (n=3)

5.2.6. Sexual health (n=3)

5.3. Social determinants (n=15)

5.3.1. Ageism (n=6)

5.3.2. Cultural diversity (n=27)

5.3.2.1. Education (n=7)

5.3.3. Environment (n=18)

5.3.3.1. Age-friendly (n=9)

5.3.3.1.1. Clinical setting (n=4)

5.3.3.1.2. Housing (n=3)

5.3.4. Financial status (n=8)

5.3.5. Gender (n=13)

5.3.6. Network

5.3.6.1. Loneliness (n=9)

5.3.6.2. Social isolation (n=14)

5.3.6.2.1. Social participation (n=8)

5.3.6.2.1.1. Intergenerational integration (n=2)

5.3.6.2.1.2. Professional activity (n=2)

6. Interventions

6.1. Health promotion (n=6)

6.1.1. Community engagement (n=5)

6.1.2. Psychosocial support (n=4)

6.1.3. Self-empowerment (n=4)

6.2. Lifestyle (n=8)

6.2.1. Nutrition (n=7)

6.2.2. Physical activity (n=9)

6.3. Vaccines (n=4)

6.3.1. Attitudes (n=3)

6.3.2. Coverage (n=2)

7. Policies (n=5)

7.1. Community engagement (n=5)

7.2. Good clinical practice (n=4)

7.3. Healthy ageing (n=5)

7.4. Inequalities (n=6)

7.5. Multisectoral partnership (n=20)

7.6. Reorientation of care (n=6)

7.6.1. Integrated care (n=6)

7.7. Voice and Meaningful Engagement

7.7.1. Caregivers (n=2)

7.7.2. Older persons (n=17)

8. Research

8.1. Evidence-based recommendations (n=11)

8.2. Implementation research (n=7)

8.3. Methodology (n=14)

8.3.1. Adaptation (n=16)

8.3.1.1. External validity (n=10)

8.3.1.1.1. International comparisons (n=4)

8.3.2. Age-disaggregated data (n=8)

8.3.3. Design

8.3.3.1. Longitudinal (n=5)

8.3.3.2. Multidisciplinary approach (n=2)

8.3.3.3. Qualitative (n=5)

8.3.4. Measurements (n=9)

8.4. Outcomes

8.4.1. Capacities (n=4)

8.4.1.1. Intrinsic capacity (n=3)

8.4.1.2. Resilience (n=2)

8.4.2. Functional ability (n=5)

8.4.3. QoL (n=14)

8.4.4. Quality of care (n=5)

8.4.5. Wellbeing (n=14)

8.4.5.1. Health and care workers (n=2)

9. Settings

9.1. Access to care (n=25)

9.1.1. Barriers (n=3)

9.2. Community (n=22)

9.3. Emergency care (n=3)

9.4. Low-resource settings (n=8)

9.4.1. Low-and-middle-income countries (n=10)

9.4.2. Rural areas (n=10)

9.5. Long-term care (n=22)

9.5.1. Framework

9.5.1.1. Data (n=3)

9.5.1.2. Standards (n=4)

9.5.2. Home care (n=3)

9.5.3. Nursing homes (n=2)

9.5.4. Palliative care (n=9)

9.5.4.1. Respite care (n=3)

9.5.5. Rehabilitation (n=4)

9.5.6. Transition care (n=4)

9.6. Primary health care (n=8)

10. Specific populations

10.1. Developmental disabilities (n=8)

10.1.1. Community engagement (n=5)

10.2. Islanders (n=6)

10.3. Isolated populations (n=6)

10.4. LGBT (n=7)

10.5. Migrants (n=10)

10.6. Minorities (n=11)

10.6.1. Indigenous (n=4)

11. Technology (n=20)

11.1. Care (n=11)

11.1.1. Loneliness (n=2)

11.1.2. Management (n=5)

11.1.2.1. Caregiver support (n=2)

11.1.3. Policies (n=2)

11.1.4. Self-care (n=2)

11.2. Digital divide (n=15)

11.2.1. Enablers (n=2)

11.3. Health promotion (n=5)

11.4. Limitations (n=2)

11.5. User-centred (n=5)

12. Training

12.1. Caregivers (n=2)

12.2. Health and care workers (n=18)

12.2.1. Communication (n=8)

12.2.1.1. Approach to minorities (n=3)

12.2.2. Prescription of lifestyle interventions (n=2)

13. Understanding the older person (n=10)

   13.1. Life experience (n=6)

   13.2. Perceptions (n=8)

   13.3. Personal values (n=7)

   13.4. Priorities (n=21)
